# The impact of the 6‐month adapted Otago Exercise Program on clinical measures of physical function relative to usual care alone in people living with dementia in residential care facilities: A pilot randomized controlled trial

**DOI:** 10.1002/alz.087435

**Published:** 2025-01-09

**Authors:** Melanie T Jones, Yanbin Dong, Haidong Zhu, Andre Soares, Charmi Patel, Colleen Hergott, Jennifer L Waller, Lufei Young, Dawnchelle Robinson‐Johnson, Richard Sams, Mark Hamrick, Deborah A Jehu

**Affiliations:** ^1^ Augusta University, Augusta, GA USA; ^2^ University of North Carolina, Charlotte, NC USA; ^3^ Claiborne Assisted Living Facility, Augusta, GA USA; ^4^ Georgia War Veterans Nursing Home, Augusta, GA USA

## Abstract

**Background:**

Dementia compromises physical function, posing risks for falls. People living with dementia (PWD) have been historically excluded from intervention trials due to researchers’ eligibility criteria. Exercise shows potential in enhancing physical function, but more evidence is needed. This pilot randomized controlled trial aimed to assess whether the adapted Otago Exercise Program improves physical function relative to usual care alone in PWD.

**Method:**

42 PWD (Montreal Cognitive Assessment<19/30) were randomized (1:1) into the exercise (n = 21) or usual care (n = 21) group at two residential care facilities [NCT05488951]. The adapted Otago Exercise Program was a physical therapist‐led strength, balance, and walking program for 1 hour, 3x/week for 6 months in groups of 5‐7 participants. Physical function was evaluated by the short physical performance battery (balance, gait speed, and chair stands), timed‐up‐and‐go (TUG), and strength at baseline and 6 months. Gait speed (m/s) was measured over 4m. The single‐task TUG involved getting up, walking 3m, walking back, and sitting down (s); the dual‐task TUG involved simultaneously performing a category task. We used dynamometers to measure leg and grip strength (kg). We performed intent‐to‐treat (ITT; n = 42) and per protocol (n = 21 usual care and n = 9 exercisers with 2/3 adherence) generalized mixed models, controlling for age, race, sex, and the MoCA.

**Result:**

For the ITT analysis, left leg strength increased in the exercise group (baseline = 13.4kg; follow‐up = 16.7kg) and decreased in the usual care group (baseline = 14.4kg; follow‐up = 13.7kg; p = 0.03). Exercise provoked faster dual‐task gait speed (baseline = 0.4m/s; follow‐up = 0.5m/s; p<0.01) and decreased TUG dual‐task duration (baseline = 37.3s; follow‐up = 27.6s; p = 0.01) with no change following usual care. The per protocol analysis showed no differences.

**Conclusion:**

The improvements in strength, gait speed, and functional mobility in the exercise group, plus the decline in strength in the usual care group suggest that exercise may prevent decline and improve physical function among PWD. There were no differences in the per protocol analysis, however, this could be due to our small sample of only n = 9 participants with ≥2/3 exercise adherence. Our results suggest that implementing any level of exercise into care plans may improve physical function in PWD, but further research should confirm these preliminary findings.

